# Filtration of Sub-3.3 nm Tungsten Oxide Particles Using Nanofibrous Filters

**DOI:** 10.3390/ma11081277

**Published:** 2018-07-25

**Authors:** Raheleh Givehchi, Qinghai Li, Zhongchao Tan

**Affiliations:** 1 Department of Mechanical & Mechatronics Engineering, University of Waterloo, Waterloo, ON N2L 3G1, Canada; raheleh.givehchi@utoronto.ca; 2 Tsinghua University—University of Waterloo Joint Research Centre for Micro/Nano Energy & Environment Technology, Tsinghua University, Beijing 100084, China

**Keywords:** air filtration, airborne nanoparticle, particle concentration, nanofibers

## Abstract

This work aims to understand the effects of particle concentration on the filtration of nanoparticles using nanofibrous filters. The filtration efficiencies of triple modal tungsten oxide (WO*_x_*) nanoparticles were experimentally determined at three different concentrations for the size range of 0.82–3.3 nm in diameter. All tests were conducted using polyvinyl alcohol (PVA) nano-fibrous filters at an air relative humidity of 2.9%. Results showed that the filtration efficiencies of sub-3.3 nm nanoparticles depended on the upstream particle concentration. The lower the particle concentration was, the higher the filtration efficiency was.

## 1. Introduction

There has been a growing interest in filtration of airborne nanoparticles over the last decade primarily due to the concerns over the potential negative impact of nanoparticles on human health and the environment [1,2,3]. Filtration of nanoparticles is used in many applications such as respiratory protection, indoor air quality, and material synthesis. While the general mechanisms of nanoparticle filtration have been well understood, disagreements exist between experiments and theoretical analyses especially for nanoparticles approaching 1 nm in diameter due to their unique properties [4,5,6,7,8,9]. Heim et al. [10] showed that the filtration efficiency of sodium chloride (NaCl) nanoparticles in the range of 2.5–20 nm followed the classical single fiber-efficiency theory. Kim et al. [11] also tested the filtration efficiency of sub-100 nm particles and presented the independency of filtration efficiency on air humidity. They also showed that the classical filtration theory agrees well with the filtration efficiency of nanoparticles down to 2 nm. However, there is a deviation for sub-2 nm particles possibly due to a thermal rebound. Boskovic et al. [12] showed the dependency of filtration efficiency of nanoparticles on the particle shape for particles ranging from 50 nm to 300 nm at the velocity of 5 cm/s in which Brownian diffusion is dominant.

Several research groups have conducted a comprehensive literature review on nanoparticle filtration that led to different conclusions. Shaffer and Rengasamy [13] concluded that the conventional filtration theory can be used for the filtration performance of respirators for particles down to 4 nm in diameter. However, Mostofi et al. [14] concluded that one of the most challenging issues in nanofiltration is the lack of knowledge on the air flow rate, the temperature and humidity, and filter life. Wang and Otani [15] reviewed recent developments on nanoparticle filtration efficiency with a focus on the effect of thermal rebound, particle shape, aggregation, flow regime, air humidity, and particle loading. In addition, Wang and Tronville [16] summarized the advances in instrumentation for the filtration of nanoparticles down to 15 nm. Givehchi and Tan [4] provided an overview on studies of nanoparticle filtration and a thermal rebound. They concluded that little was known in this area of research.

While various studies have been completed on the filtration of airborne nanoparticles, limited data have been published to date on the effects of particle concentration on nanoparticle filtration efficiency. Understanding the behavior of nanoparticles down to 1 nm requires information on size distribution and composition of small nanoclusters [17]. According to conventional filtration theories, the filtration efficiency of particulate matter is independent of particle concentration. However, this was not validated for very small nanoparticles, which usually have a high number of concentrations. Ardkapan et al. [18] investigated the effect of particle concentration on the removal efficiency of an electrostatic fibrous filter. According to this research, nanoparticles up to 7 nm with a high concentration are captured with a higher rate due to the electrostatic forces rather than those with a low concentration. This knowledge is expected to be important for the design of high efficient filters, but it is missing in literature.

The objective of this study is to understand the effects of nanoparticle concentration on nanoparticle filtration. In the following section, the filtration efficiencies of WO*_x_* particles ranging from 0.82 nm to 3.3 nm for triple modal number concentration distributions were experimentally determined. Experiments were conducted at three different particle concentrations using PVA nanofibrous filters at the relative humidity of about 2.9%.

## 2. Materials and Experimental Methods

### 2.1. Materials

**Figure 1:**
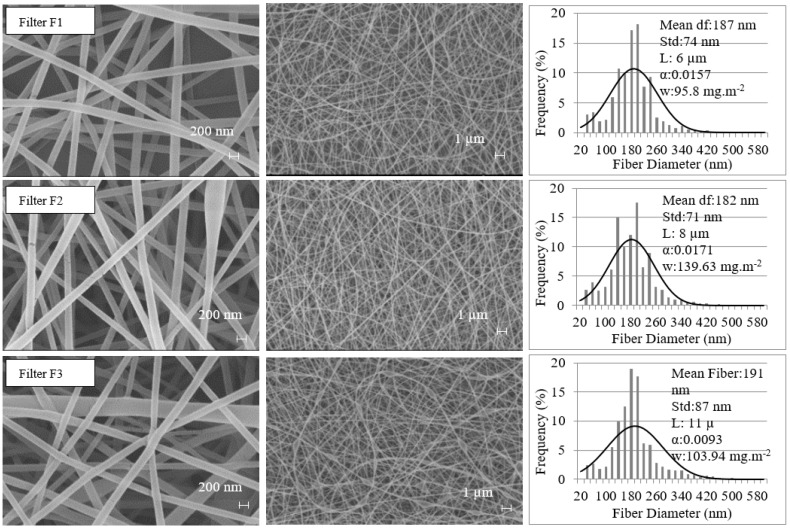

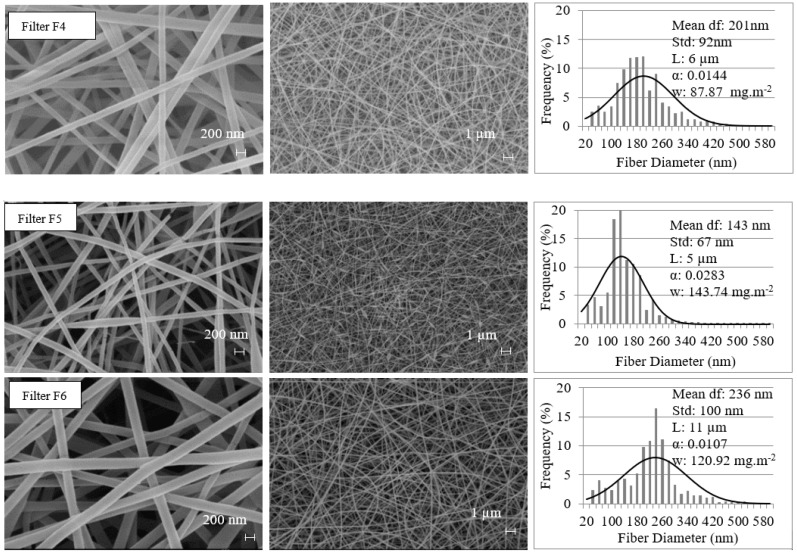
SEM images and fiber diameter distributions of nanofibrous filters.

Electrospun PVA nanofibrous filters made in a laboratory setting were used as the test filters. The characteristics of these nanofibrous filters have been described in other studies [19]. The solidity of filters is determined based on the measured pressure drop and filter thickness using the Davies equation [20]. Differences in the electrospinning parameters result in different mean fiber diameters, thicknesses, and solidities of the filters. Figure 1 shows the scanning electron microscope (SEM) images (Zeiss Gemini Model Leo 1550, Feldbach, Switzerland), which were taken at two magnifications, × 20K and × 5K, and fiber specifications of the sample.

### 2.2. Experimental Methods

**Figure 2:**
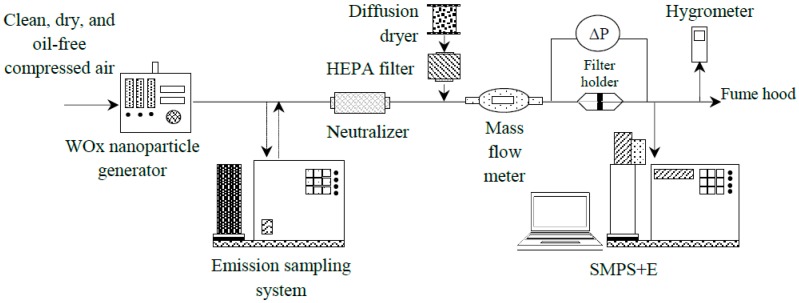
Schematic diagram of the experimental setup.

As shown in Figure 2, the experimental setup used to determine the filtration efficiency of WO*_x_* nanoparticles is similar to the one introduced in our previous publication [5] with an additional aerosol dilution system.

Nanoparticles down to 0.8 nm in diameter were generated using the WO*_x_* nanoparticle generator (Model 7.860, GRIMM Aerosol techniK, Ainring, Germany). The size distribution remained stable during the experiments. The WO*_x_* aerosol flow rate flow rate ranged from 7 L·h^−1^ to 10 L·h^−1^. The carrier air flow rate ranged from 120 L·h^−1^ to 200 L·h^−1^. The diluting air flow ranged from 200 L·h^−1^ to 250 L·h^−1^. Since the diffusion loss might be significant for such small particles, particle concentrations have to be large enough to be sufficient when the aerosol flow reaches the downstream measurement device. To detect these small particles, the aerosol flow rates were relatively high (4 lpm). A high flow rate is expected to shorten the travelling time of nanoparticles in the tubes and to reduce the diffusion loss of nanoparticles [21]. The relative humidity and temperature of the air flow during the experiments were 2.9 ± 0.4% and 24.7 ± 1.3 °C, respectively. This relative humidity does not represent real world nanoparticles. However, it was chosen because the particle generator generated nanoparticles at this small relative humidity. This low relative humidity is deemed to minimize the capillary force and minimize the adhesion energy between nanoparticles and filter fibers due to a capillary force [5].

The air emission sampling system (ESS, Model 7.917, GRIMM Aerosol techniK, Ainring, Germany) was employed to dilute the nano-aerosol for tests at lower concentrations. The dilution air and original aerosol were mixed in a counter flow mixer before passing through the aerosol cooler to reach room temperature. The dilution system is expected to prevent the condensation on nanoparticles and the formation of new nanoparticles. It also reduces the sample temperature to a level that is required by the measurement device. At the sample flow rate of 1 lpm, the dilution ratios were 1:10 and 1:100 for the first and second dilution stages, respectively. Note that particles travel in extra tubes due to the introduction of the ESS, which may further lower the particle concentration. Nanoparticles that survived were then passed through a radioactive neutralizer (Model P-2031, Staticmaster, Cincinnati, OH, USA), which was followed by the test filters. 

The scanning mobility particle sizer coupled with a Faraday cup electrometer (SMPS+E, Model 5.706, GRIMM Aerosol techniK, Ainring, Germany) consists of a differential mobility analyzer (DMA, GRIMM Aerosol techniK). A Faraday cup electrometer (FCE, Model 5.705, GRIMM Aerosol techniK, Ainring, Germany) was used to measure nanoparticle size distribution before and after filtration. From previous studies, we are aware of the fact that many factors may lead to artifacts in the measured results. One of the most important issues associated with measuring particles in small sizes are high diffusion losses and low charging probability [22]. The resolution of DMA could be reduced by diffusing the broadening of small nanoparticles [23]. Some researchers related diffusion broadening to the observation of thermal rebound in relatively early publications [24,25,26,27]. Their experimental results were later challenged because of the accuracy of the DMA.

With this in mind, we attempted to minimize this kind of artifact. The first approach is to choose a Faraday cup electrometer over a condensation particle counter (CPC) as the particle detector downstream of the DMA. One critical challenge for CPC is that a high diffusional loss causes low counting efficiency for very small nanoparticles. The lower particle size detection limit of a commercial CPC is approximately 2 nm [28], which recently decreased to about 1 nm [29,30] and it is sensitive to the operating condition, the particle composition, and the charge state [29,31,32,33].

As an alternative device for detecting nanoparticles, FCE detects the charged nanoparticles at a response time of less than 100 ms [34] and it was believed to precisely detect nanoparticles of various compositions [28]. The lower detection limit of an FCE depends on the sensitivity of the specific FCE. GRIMM FCE was employed as a reference device for particles smaller than 3 nm [29] and FCE demonstrated a higher accuracy for smaller particles than CPC. Furthermore, FCE works well in high concentration samples [22]. For all these reasons, the GRIMM SMPS+E with short-DMA (S-DMA, GRIMM Aerosol techniK, Model 5.706) was employed in this research. It was capable of sizing and quantifying nanoparticles down to 0.8 nm. Furthermore, a sheath air to sample airflow ratio of 20:2 was used and remained constant in all experiments in order to minimize the diffusion loss of particles.

Both monodispersed and poly-dispersed nanoparticles have been used for filtration tests in literature. Some researchers employed monodispersed particles classified by a DMA [10,25,27,35,36,37,38,39,40]. Others used poly-dispersed particles and measured the particle number concentrations with an SMPS [41,42,43,44,45,46,47,48,49,50,51,52,53]. Possible errors when poly-dispersed particles are used can be eliminated by using a proper sampling method either by employing a time interval [48] or by introducing purge time [54] between measurements for an upstream and a downstream particle number concentration distribution. Both upstream and downstream concentrations reach equilibrium between consecutive sampling. Furthermore, a recent study confirmed that passing poly-dispersed nanoparticles and monodispersed nanoparticles through identical filters resulted in the same particle penetration measurement [55]. In the current study, therefore, the FCE and the DMA were set apart from each other at a minimum distance to reduce the nanoparticle loss due to diffusion. In our experiments, the particle size distribution measurements were stable as long as the particle concentrations were greater than 1000 particles/cm^3^.

## 3. Results and Discussion

### 3.1. Filtration Efficiencies of Tested Filters for Sub-3.3 nm Particles

**Figure 3:**
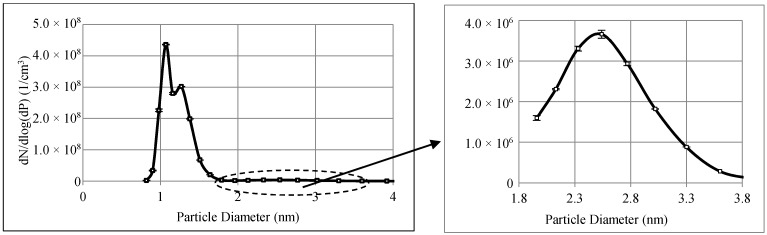
WO*_x_* nanoparticle size distribution generated by the tungsten oxide generator at an aerosol flow rate of 4 lpm.

Figure 3 shows the size distribution of the WO*_x_* nanoparticles generated using the nano-aerosol generator. Each data point is averaged over at least three repeated measurements. The corresponding aerosol flow rate was 4 lpm. The tungsten air, the carrier air, and the diluting air flow rates in the tungsten aerosol generator were 10 L·h^−1^, 220 L·h^−1^, and 250 L·h^−1^, respectively. The particle number concentrations of these particles remained stable.

As shown in Figure 3, the generated nanoparticles ranged from 0.82 nm to 4 nm in diameter. There are three peaks corresponding to 1.07 nm, 1.27 nm, and 2.54 nm. The particle concentrations for the first two peaks are in the order of 10^8^ particles/cm^3^ while the third peak is about 100 times lower than the other two (in the order of 10^6^ particles/cm^3^). 

**Figure 4:**
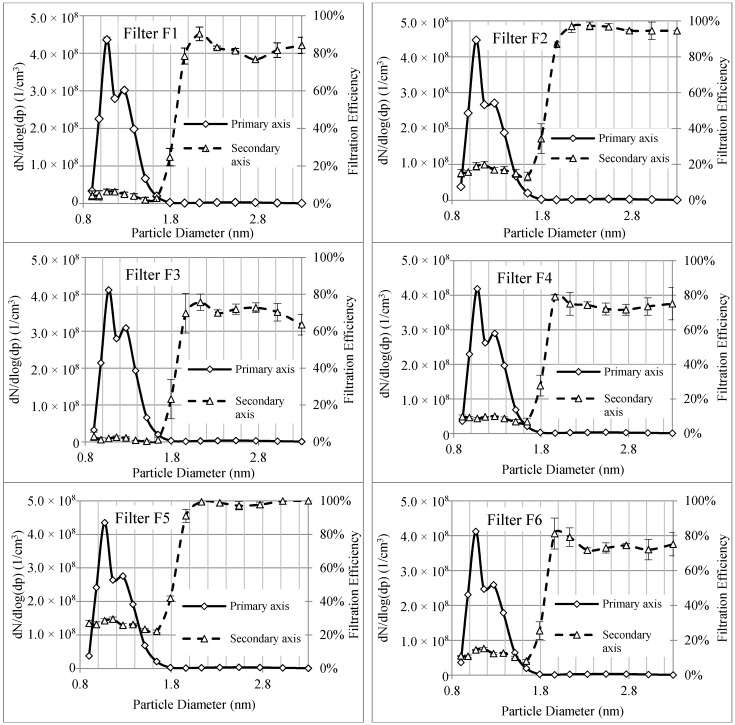
Nanoparticle size distributions along with particle removal efficiencies of different nanofibrous electrospun filters.

Figure 4 shows the upstream particle concentrations and the corresponding filtration efficiencies for six different electrospun nanofibrous filters. While the particle sizes ranged from 0.82 nm to 4 nm or more, the filtration efficiency was determined only for particles that ranged from 0.9 nm to 3.3 nm. This is because the particle concentrations of both lower and upper ends dropped below the lower detection limit of FCE in the air downstream of the filters. Comparing the filtration efficiencies for particles ranging from 0.9 nm to 3.3 nm for different tested filters shows that the measured filtration efficiencies depended on the concentrations of these nanoparticles. The measured filtration efficiencies for nanoparticles smaller than 1.96 nm are much lower than those for larger ones and there is a sharp drop in filtration efficiency when particle concentration increased as size decreased. Particle concentrations for sub-1.96 nm particles are in the range of 10^8^ particles/cm^3^. The concentrations of larger particles are around 10^6^ particles/cm^3^. The difference between the filtration efficiencies for these particles may be attributed to the differences in the incoming particle concentrations.

Further investigations show that the concentration of particles with a diameter of 1.79 nm is about the same as those with diameters of 2.77 nm and 2.33 nm. However, the filtration efficiency for 1.79 nm particles is much lower than the filtration efficiency of those with the other two diameters. In addition to particle concentrations, another mechanism may also contribute to the drop in filtration efficiency for smaller sized particles.

According to the conventional filtration model, the filtration efficiency increases as the size of small nanoparticles decreases [56]. However, results from this study showed clear drops in filtration efficiencies when the size of nanoparticles were below 1.96 nm. It appears that the conventional filtration model may need modification for these tested sizes of WO*_x_* particles through the PVA nanofibrous filters and that the effect of particle concentration may have to be introduced into the models. 

### 3.2. Effects of Particle Concentration on the Filtration of Nanoparticles

To systematically investigate the effects of nanoparticle concentration on air filtration, the ESS was employed to dilute the incoming aerosol prior to filtration tests. Since the original concentrations of particles larger than 1.96 nm in diameter were in the order of 10^6^ particles/cm^3^ (see Figure 3). The concentrations of large particles after dilution approached the detection limit of the FCE. Therefore, the effects of particle concentration on filtration efficiency are only presented in this study for sub-1.8 nm nanoparticles.

**Figure 5:**
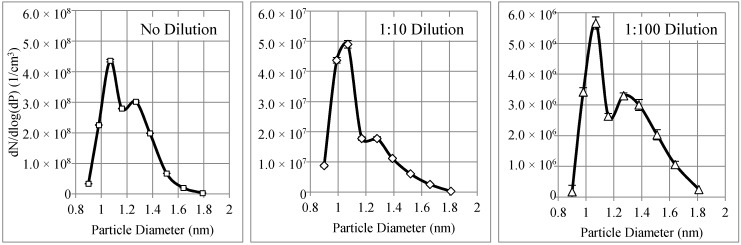
WO*_x_* nanoparticle number concentration distributions generated by the tungsten oxide generator at an aerosol flow rate of 4 lpm.

Three particle number concentration distributions, which correspond to no dilution, were diluted 10 times (1:10) and 100 times (1:100). These concentration distributions are shown in Figure 5. The particle concentrations have three different orders of magnitude from 10^8^ to 10^7^ and 10^6^ particles/cm^3^. The error bars in terms of standard deviation in the particle size range were less than 1% for a high particle concentration (in the order of 10^8^), about 3% for the median (in the order of 10^7^), and about 4% for a low particle concentration (in the order of 10^6^). In all three figures, there are two peaks at 1.07 nm and 1.27 nm. 

**Figure 6:**
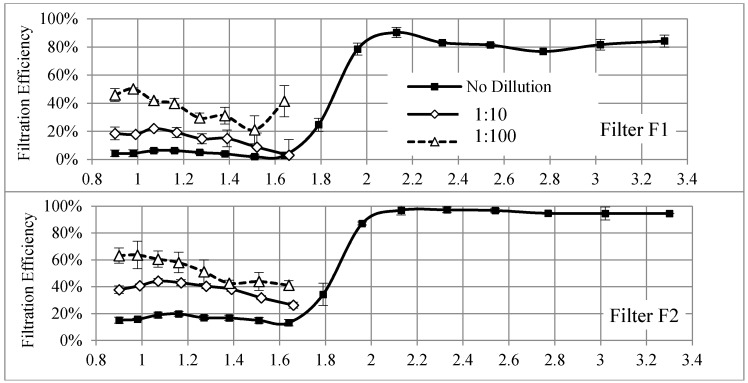

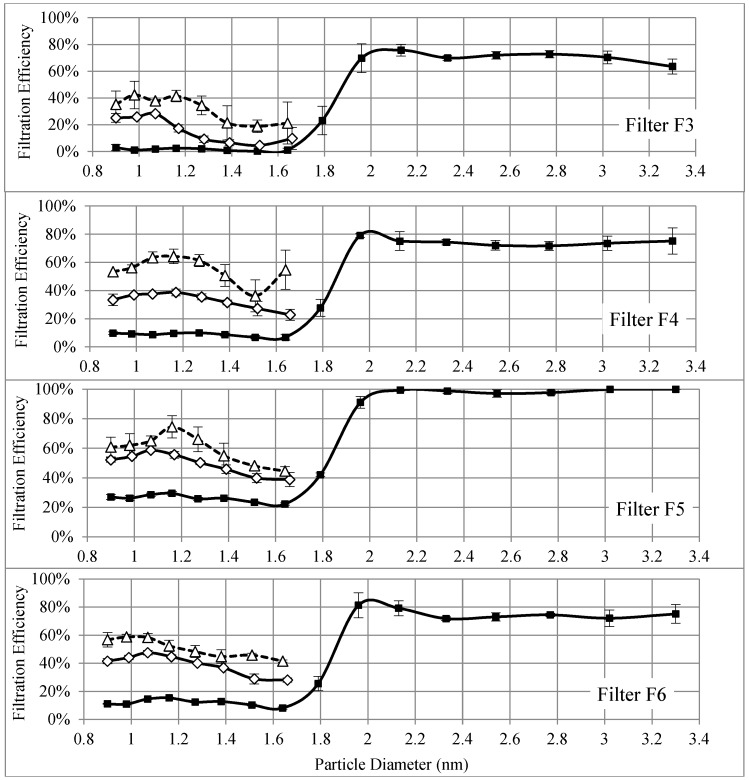
Particle filtration efficiencies of nanofibrous filters at three levels of particle concentrations (no dilution, 1:10 dilution, and 1:100 dilution).

Figure 6 shows the measured filtration efficiencies of the electrospun nanofibrous filters for three cases from Figure 5. The results show clear dependencies of filtration efficiencies that rely on the incoming (upstream) particle concentration. The filtration efficiency increased with the dilution ratio. This observation is similar to the results reported by Shi and Ekberg (2015) except that the particle sizes were larger in their work. They also showed that the filtration efficiencies of particulate matter ranging from 300 nm to 500 nm decreased as the upstream particle concentration increased [57].

The upstream particle concentrations for sub-1.8 nm particles when employing the second dilution stage of ESS are in the same magnitude as the concentrations in larger particles. Even at the same concentration level, the filtration efficiencies for sub-1.8 nm particles are lower than the filtration efficiencies of larger particles for all tested filters.

**Figure 7:**
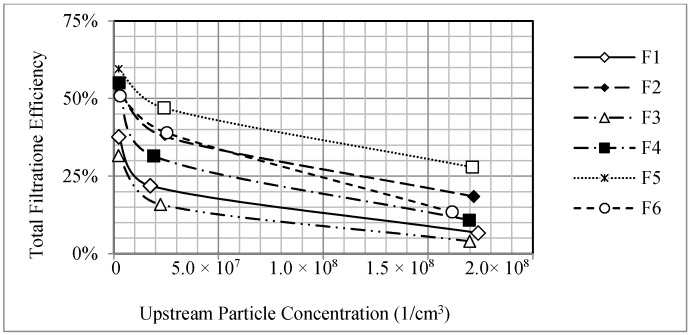
Total filtration efficiencies of electrospun filters as a function of upstream particle concentration.

Figure 7 shows the filtration efficiencies for sub-1.8 nm particles as a function of upstream particle concentration for different nanofibrous filters. It was found that the measured filtration efficiencies also decreased with increasing particle concentration. Filter F5 showed the highest filtration efficiencies and Filter F3 showed the lowest filtration efficiencies for sub-1.8 nm particles at the three particle concentrations. Even though Filter F5 has the least thickness of 5 µm, it has the smallest mean fiber diameter (143 nm) and the greatest solidity (0.0283). The larger solidity to the mean fiber diameter ratio is likely to increase the filtration efficiency for nanoparticles at the price of a relatively large pressure drop of 228.93 Pa. Filter F3 has a relatively large thickness of 11 µm, which is thicker than other filters. Its mean fiber diameter is between those of Filters F1 and F4. However, it has the least solidity among all the filters, which decreases its filtration efficiency. The pressure drop of this filter is 42.30 Pa, which is lower than the pressure drop of other filters. Therefore, for these sizes of nanoparticles, which might behave like large gas molecules, a filter with a smaller mean fiber diameter and larger solidity has the highest filtration efficiency while the thickness of the filter has a negligible effect on the filtration efficiency among these nanoparticles.

### 3.3. Discussion on Thermal Rebound

Figure 6 also showed that the filtration efficiencies for particles smaller than 1.07 nm to 1.17 nm decreased as the size of nanoparticles decreased. This finding led us to revisit a possible thermal rebound that remains a debatable subject in literature. As explained above, nanofibrous filters and sub-1.8 nm WO*_x_* particles were used in this study. In addition, all the filtration tests were conducted at a relatively low humidity. These parameters are different from other previous studies in thermal rebound. The majority of them could not experimentally prove the thermal rebound of nanoparticles. So far, only three studies have shown certain results of thermal rebound. Kim et al. [11] observed a drop in the filtration efficiency of a glass filter for uncharged sub-1.3 nm NaCl nanoparticles at a relative humidity of 1.22%. Van Gulijk and Schmidt-Ott [58] compared the penetration of different particles with a similar size distribution through a wire screen and found the possibility of thermal rebound for NaCl and NiSO_4_ particles. However, they did not determine the particle critical diameter below which thermal rebound occurred. Rennecke and Weber [59] then investigated the thermal rebound of nanoparticles under low pressure and demonstrated the possibility of thermal rebound for dense NaCl particles ranging from 20 nm to 60 nm. They proposed that the thermal rebound was pressure dependent while, under normal conditions, gas friction may reduce the kinetic energy of a particle prior to rebounding and may cause its adhesion to a filter media surface.

We believe that the properties of filter media may also be important for the occurrence of thermal rebound. Most previous experimental studies employed commercial fibrous filters to test nanoparticle penetration through the filters, which led to conclusions that no thermal rebound was associated with their tested particles [37,47,52,54,60]. Thermal rebound may not be observed for thick and multilayer filters because rebounded particles can be captured by other fibers within the thick filter [4]. Consequently, single layer filters such as wire screens and thin nanofibrous filters are expected to minimize this artifact.

Two other earlier experimental studies showed the possibility of thermal rebound for sub-2 nm particles and they used wire screens [26,27]. However, wire screens have well—defined wires and openings. These open spaces may be sufficiently large for nanoparticles to pass through without collision on the wires. Therefore, the low efficiency of small nanoparticles may have been attributed inaccurately to a thermal rebound.

In the study, the tested nanofibrous filters are extra thin (L < 10 µm) and act like a single layer media. The media surface on which nanoparticles can collide is greatly increased due to the large surface area of nanofibers, which increases the possibility of thermal rebound if it does exist. If a particle rebounds from the nanofiber, there will be little chance for it to be captured again by other nanofibers. The filtration is then characterized by surface loading instead of depth loading because of the extra thin thickness of nanofibers.

Other important factors that may affect thermal rebound are the properties of nanoparticles. Although various methods have been used to produce test nano-aerosol for experimental studies of thermal rebound, very few can generate nanoparticles down to 1 nm with sufficiently high concentration. This was mainly limited by the availability of devices. Earlier studies that tested only nanoparticles greater than 2 nm did not show thermal rebound. Among a handful of papers where sub-2 nm particles were tested along with larger ones [11,25,26,27,30], three studies reported the possibility of thermal rebound for sub-2 nm particles [11,26,27]. Employing the sub-2 nm WO*_x_* particles in the current study may be one of the factors that led to the observation of possible thermal rebound. 

Several studies mentioned a charge effect as a reason why thermal rebound has not been observed yet. Particles are assumed to be neutral in the thermal rebound theory. However, charged particles induce an image force and reduce the rebound probability [30,58]. Heim et al. [30] investigated penetration of particles down to 1.2 onto wire grids. Based on this study, for the charged particles of WO_x_ and tetra-heptyl ammonium bromide, no thermal rebound was observed and the lower penetration of particles for a smaller particle is due to a small contribution by the image charge effect coupled with the diffusion. The generated nanoparticles in the current study are highly charged due to the effect of the thermal emission of electrons in the ceramic tube [30,61]. The majority of charged particles lost their charge in the neutralizer. However, a small fraction of charged particles remained and affected the results of thermal rebound.

The low relative humidity in this study may have also contributed to the observation of thermal rebound if it is the case. First, the adhesion energy between particles and filter media surfaces increased with the level of relative humidity due to the capillary force [5,62,63,64,65]. The increase in the adhesion energy may decrease the probability of thermal rebound.

The results herein showed that the critical diameters for thermal rebound were nearly the same for various dilution ratios. However, the drop-in filtration efficiencies (implying possible thermal rebound) is more obvious at lower particle concentrations (i.e., higher dilution ratios). This is also deemed reasonable. At lower particle concentrations, individual particles have more chances to collide with the nanofibers, which increases the probability of nanoparticle rebound from the surface and decreases the adhesion efficiency of particles to the surface.

There might be another hypothesis regarding the effect of particle concentration on thermal rebound. The high filtration efficiencies for nanoparticles at low concentrations likely resulted from the increased availability of the filtration surface area. This may be similar to the process of gas adsorption, which is concentration-dependent. Adsorption is a surface phenomenon caused by van der Waals forces [66] where gas molecules may stay on the surface of an adsorbent. In this process, as the concentration of gas molecules increases, more surfaces of solid are covered with gas molecules. Therefore, the availability of surface area decreases at higher concentrations [67]. It has been well accepted that nanoparticles behave differently than micron ones. Nanoparticles at these extremely small sizes (sub-1.8 nm) may behave like gas molecules or molecular clusters when they collide with the surface of the filter media. To quantify this concentration dependency, a mechanism that might be similar to adsorption can be considered for these extremely small nanoparticles.

All of the aforementioned factors may have led to a decreased rate of filtration efficiency for particles smaller than the critical diameters. The critical particle diameters are also almost constant for the differently tested nanofibrous filters. Since all tested filters were made of the same materials with the same mechanical constants (e.g., Hamaker constant, elastic mechanical constant, and specific adhesion energy), one would expect the particle critical diameter to be the same for the filtration of WO*_x_* particles using PVA filters.

It would be useful to employ thin fibrous filters with micron scale fibers and determine if the same phenomenon occurs. Micron-scale fibers have a lower surface area to volume ratio than nano-scale fibers and it is expected that particles would cover more surfaces. Therefore, based on the initiative model proposed above, one would expect to observe similar trends for filters made of micron fibers. Furthermore, a systematic investigation by various types of particles and filters would lead to a better understanding in this subject. Additionally, considering the effect of particle bouncing and resuspension may also improve the results [68].

## 4. Conclusions

To summarize, this study showed that sub-3.3 nm WO*_x_* particles smaller and larger than 1.96 nm behaved differently in air filtration. Filtration efficiencies dropped for particles with high particle concentrations. This study provides evidence of the existence of thermal rebound. For particles ranging from 1.07 nm to 1.17 nm, the reduction in filtration efficiency may be a result of thermal rebound. This reduction is more clear at lower particle concentrations because nanoparticles have more chance to collide with the surface of filter media.
